# Supplementary material for: Playing games with multiple access channels
Source: Nat Commun. 2020 Mar 20;11:1497. doi: 10.1038/s41467-020-15240-w (PMC7083904; doi:10.1038/s41467-020-15240-w)
Supplement: Supplementary file 1 — Supplementary Information [file 41467_2020_15240_MOESM1_ESM.pdf]

Supplementary Information:
Playing Games with Multiple Access Channels

Leditzky et al.

Supplementary Note 1. Preliminaries

1.1 Non-local Games

A two-player non-local game is a tuple $G = (\mathcal{X}_1, \mathcal{X}_2, \mathcal{Y}_1, \mathcal{Y}_2, P, W)$, where $\mathcal{X}_i$ and $\mathcal{Y}_i$ are the question and answer sets for player $i = 1, 2$, respectively. The set $P \subset \mathcal{X}_1 \times \mathcal{X}_2$ is called the *promise* of the game, the set of "allowed" questions. The set $W \subset P \times \mathcal{Y}_1 \times \mathcal{Y}_2$ is the *winning condition*, i.e., upon receiving the questions $(x_1, x_2) \in P$ and answering with $y_i \in \mathcal{Y}_i$, the players win the game if $(x_1, x_2, y_1, y_2) \in W$, and lose otherwise.

Every game $G = (\mathcal{X}_1, \mathcal{X}_2, \mathcal{Y}_1, \mathcal{Y}_2, P, W)$ with promise $P \subsetneq \mathcal{X}_1 \times \mathcal{X}_2$ can be turned into a *promise-free* game $G' = (\mathcal{X}_1, \mathcal{X}_2, \mathcal{Y}_1, \mathcal{Y}_2, P', W')$ by declaring $P' = \mathcal{X}_1 \times \mathcal{X}_2$ and $W' = W \cup (P^c \times \mathcal{Y}_1 \times \mathcal{Y}_2)$, i.e., the players win automatically if they receive a question pair outside the promise P. We write $G = (\mathcal{X}_1, \mathcal{X}_2, \mathcal{Y}_1, \mathcal{Y}_2, W)$ for a promise-free game.

While the two players Alice and Bob can agree on a strategy beforehand, they are not allowed to communicate during the game. A *deterministic strategy* for Alice and Bob is a pair of deterministic functions $f_i \colon \mathcal{X}_i \to \mathcal{Y}_i$ for $i = 1, 2$. A *probabilistic strategy* for Alice and Bob is a probabilistic mixture of deterministic strategies. For a given probability distribution $\pi \colon P \to [0, 1]$ on the promised question set $P \subset \mathcal{X}_1 \times \mathcal{X}_2$, we define $\omega(G, \pi)$ as the maximal winning probability using probabilistic strategies. If the given probability distribution on P is the uniform distribution π_U, we use the shorthand $\omega_U(G) \equiv \omega(G, \pi_U)$. Note that the maximal winning probability is always achieved on an extremal point, i.e., a deterministic strategy. A strategy achieving $\omega(G, \pi) = 1$ is called *perfect*.

1.2 Capacity Region of Multiple Access Channels

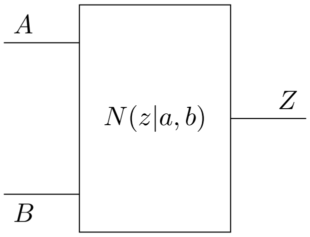

Supplementary Fig. 1: Mathematical model of a multiple access channel.
The two senders and the single receiver of a multiple access channel $N(z|a, b)$ are associated with random variable A, B and Z, respectively.

In this paper we only consider discrete memoryless multiple access channels without feedback, to which we refer simply as multiple access channels (MAC). A two-sender MAC is a tuple $(\mathcal{A}, \mathcal{B}, \mathcal{Z}, N(z|a, b))$, where $\mathcal{A}$ and $\mathcal{B}$ are the input alphabets for sender 1 and 2, respectively, $\mathcal{Z}$ is the output alphabet for the single receiver, and $N(z|a, b)$ is a probability distribution for all pairs $(a, b) \in \mathcal{A} \times \mathcal{B}$ (see Supplementary Fig. 1).

The following discussion is taken from [1]. An $(R_1^{(n)}, R_2^{(n)}, n)$-code is a tuple $(\mathcal{M}_1, \mathcal{M}_2, a^n, b^n, \hat{z}^n)$, where:

- $\mathcal{M}_1$ and $\mathcal{M}_2$ are message sets with $|\mathcal{M}_i| = 2^{nR_i^{(n)}}$ for $i = 1, 2$;
- $a^n \colon \mathcal{M}_1 \to \mathcal{A}^n$ and $b^n \colon \mathcal{M}_2 \to \mathcal{B}^n$ are encoding functions;
- $\hat{z}^n \colon \mathcal{Z}^n \to \mathcal{M}_1 \times \mathcal{M}_2 \cup \{e\}$ is a decoding function, with e an arbitrary error message.

Without loss of generality we assume a uniform distribution over the messages (M_1, M_2); in particular, the codewords $a^n(M_1)$ and $b^n(M_2)$ are independent. The average probability of error is defined as

$$P_e^{(n)} \coloneqq \Pr\{(\hat{M}_1, \hat{M}_2) \neq (M_1, M_2)\}, \tag{1}$$

where $(\hat{M}_1, \hat{M}_2)$ is a random variable with probability mass function (pmf) $\hat{z}^n(N^{\times n}(z^n|a^n(M_1), b^n(M_2)))$. A rate pair (R_1, R_2) is said to be achievable if there exists a sequence of codes $\{(R_1^{(n)}, R_2^{(n)}, n)\}_{n\in\mathbb{N}}$ such that $\liminf_{n\to\infty} R_i^{(n)} = R_i$ and $\lim_{n\to\infty} P_e^{(n)} = 0$. The capacity region $\mathcal{C}(N)$ of the MAC $(\mathcal{A}, \mathcal{B}, \mathcal{Z}, N(z|a, b))$ is the closure of the set of all achievable rate pairs (R_1, R_2). We also consider the sum-capacity $S(N)$ defined as $S(N) \coloneqq \sup\{R_1 + R_2 \colon (R_1, R_2) \in \mathcal{C}(N)\}$.

The capacity region $\mathcal{C}(N)$ of a two-sender MAC $(\mathcal{A}, \mathcal{B}, \mathcal{Z}, N(z|a, b))$ has a single-letter characterization [2, 3]. Let (A, B) be a pair of discrete random variables jointly distributed according to the pmf $p_A p_B$, and let Z be the channel output random variable with conditional pmf $N(z|a, b)$. Define $\mathcal{R}(A, B)$ as the set of rate pairs (R_1, R_2) satisfying

$$\begin{aligned} R_1 &\leq I(A; Z|B) \\ R_2 &\leq I(B; Z|A) \\ R_1 + R_2 &\leq I(A, B; Z). \end{aligned} \tag{2}$$

Then the capacity region $\mathcal{C}(N)$ of the MAC $(\mathcal{A}, \mathcal{B}, \mathcal{Z}, N(z|a, b))$ is the convex hull of the union of the regions $\mathcal{R}(A, B)$ over all product distributions $p_A p_B$:

$$\mathcal{C}(N) = \operatorname{conv}\left(\bigcup \{\mathcal{R}(A, B) \colon (A, B) \sim p_A p_B\}\right) \tag{3}$$

1.3 MACs and Entanglement Assistance

In this paper we consider coding strategies for classical MACs assisted by entanglement shared between the two senders.[1] To formalize this setting, let $(\mathcal{A}, \mathcal{B}, \mathcal{Z}, N(z|a, b))$ be a MAC as defined in Section 1.2, and let the two senders A and B share an entangled state $|\psi\rangle_{S_A S_B} \in \mathbb{C}^d \otimes \mathbb{C}^d$, where the d-dimensional quantum systems S_A and S_B with $|S_A| = |S_B| = d$ are with senders A and B, respectively. We then consider the following coding scenario.

Let A_1 and B_1 be random variables taking values in finite alphabets $\mathcal{A}_1$ and $\mathcal{B}_1$ for sender A and B, respectively. Depending on the value $a_1 \in \mathcal{A}_1$ of A_1, the first sender selects a positive operator-valued measure (POVM) $L^{(a_1)} = \{L_{a_2}^{(a_1)}\}_{a_2\in\mathcal{A}_2}$ with

$$L_{a_2}^{(a_1)} \geq 0 \quad \text{for all } a_1 \in \mathcal{A}_1,\ a_2 \in \mathcal{A}_2 \text{ and} \tag{4}$$

$$\sum_{a_2\in\mathcal{A}_2} L_{a_2}^{(a_1)} = I_d \quad \text{for all } a_1 \in \mathcal{A}_1. \tag{5}$$

Here, $\mathcal{A}_2$ is some finite alphabet, and I_d denotes the identity operator on $\mathbb{C}^d$. Likewise, for $b_1 \in \mathcal{A}_1$ the second sender selects a POVM $M^{(b_1)} = \{M_{b_2}^{(b_1)}\}_{b_2\in\mathcal{B}_2}$ for some finite alphabet $\mathcal{B}_2$ satisfying $M_{b_2}^{(b_1)} \geq 0$ for all $b_1 \in \mathcal{B}_1$, $b_2 \in \mathcal{B}_2$ and $\sum_{b_2\in\mathcal{B}_2} M_{b_2}^{(b_1)} = I_d$ for all $b_1 \in \mathcal{B}_1$. Upon drawing (a_1, b_1), the senders measure their respective half of the entangled state $|\psi\rangle_{S_A S_B}$ using the measurements $L^{(a_1)}$ and $M^{(b_1)}$, producing a correlation

$$P(a_2, b_2|a_1, b_1) = \langle\psi| L_{a_2}^{(a_1)} \otimes M_{b_2}^{(b_1)} |\psi\rangle. \tag{6}$$

[1] We briefly discuss entanglement assistance where each sender shares entanglement with the receiver at the end of Section 3.3.

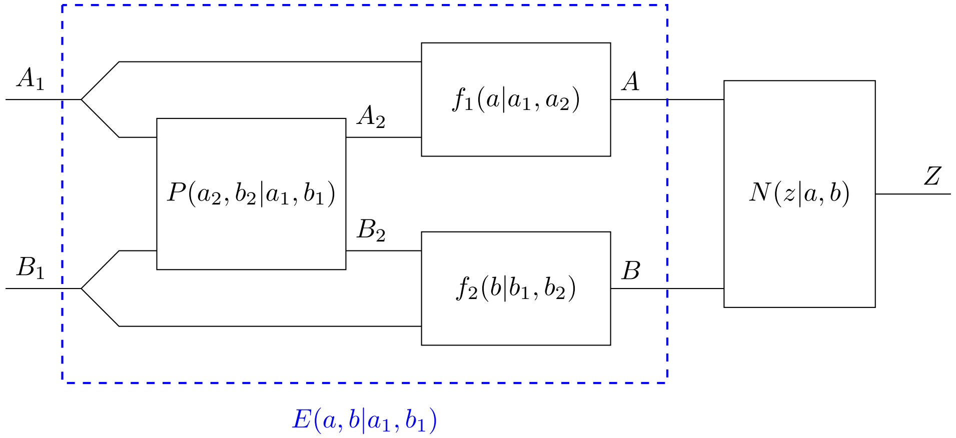

Supplementary Fig. 2: Entanglement-assisted coding scenario for a multiple access channel. The encoding E (blue rectangle) is composed of the following: a correlation P obtained from each sender measuring her system of a shared entangled state with POVMs selected according to the inputs A_1 and B_1 (see (6)), and a post-processing of the outcomes a_2 and b_2 together with a_1, b_1 using functions f_i to obtain the inputs a and b to the MAC N. If A_1 and B_1 are independent random variables, then the total channel $N \circ E$ can be interpreted as a MAC with input A_1 and B_1 and output Z, whose capacity region $\mathcal{C}(N \circ E)$ (as defined in (3)) is equal to the d-entanglement-assisted achievable rate region $\mathcal{C}^{(1)}_{\mathrm{ea},d}(N)$ defined in (8).

Finally, the senders can each post-process their measurement outcomes a_2 and b_2 together with their inputs a_1 and b_1 to produce inputs a and b to the MAC N, which we summarize in a function $f_1(a|a_1, a_2)f_2(b|b_1, b_2)$.[2] In total, we have the classical channel

$$E(a, b|a_1, b_1) = f_1(a|a_1, a_2)f_2(b|b_1, b_2)P(a_2, b_2|a_1, b_1), \tag{7}$$

where the correlation P is obtained from measuring the shared entangled state $|\psi\rangle_{S_A S_B}$ through (6). The setup is depicted in Supplementary Fig. 2.

If we require the senders to draw a_1 and b_1 independently from a product distribution $p_{A_1}(a_1)p_{B_1}(b_1)$, then the channel $N \circ E$ with E as defined in (7) can again be interpreted as a MAC with input alphabet $\mathcal{A}_1 \times \mathcal{B}_1$ and output alphabet $\mathcal{Z}$. This prompts us to define the *d-entanglement-assisted achievable rate region* of a classical MAC N as

$$\mathcal{C}^{(1)}_{\mathrm{ea},d}(N) \coloneqq \bigcup_E \{\mathcal{C}(N \circ E)\}, \tag{8}$$

where $\mathcal{C}(\cdot)$ is the capacity region of an ordinary MAC defined in (3), and the union is over all classical channels E as in (7) defined in terms of the following data:

- an entangled state $|\psi\rangle_{S_A S_B} \in \mathbb{C}^d \otimes \mathbb{C}^d$;
- arbitrary finite alphabets $\mathcal{A}_1, \mathcal{B}_1$ and $\mathcal{A}_2, \mathcal{B}_2$;
- POVMs $L^{(a_1)} = \left\{L^{(a_1)}_{a_2}\right\}_{a_2 \in \mathcal{A}_2}$ for $a_1 \in \mathcal{A}_1$ and $M^{(b_1)} = \left\{M^{(b_1)}_{b_2}\right\}_{b_2 \in \mathcal{B}_2}$ for $b_1 \in \mathcal{B}_1$, defined on $\mathbb{C}^d$;

[2]Note that in principle this post-processing can be made part of the measurements with a potential increase of the local dimension d. However, we choose to keep them separate in order to link the local dimension d to the non-local games considered in Section 4 in a clean way.

- post-processings $f_1\colon \mathcal{A}_1 \times \mathcal{A}_2 \ni (a_1, a_2) \mapsto a \in \mathcal{A}$ and $f_2\colon \mathcal{B}_1 \times \mathcal{B}_2 \ni (b_1, b_2) \mapsto b \in \mathcal{B}$

We also define the corresponding *achievable sum rate*

$$S_{\mathrm{ea},d}(N) \coloneqq \sup\{R_1 + R_2 \colon (R_1, R_2) \in \mathcal{C}^{(1)}_{\mathrm{ea},d}(N)\}. \tag{9}$$

The coding theorem (3) for unassisted MACs implies that the region $\mathcal{C}^{(1)}_{\mathrm{ea},d}(N)$ in (8) is achievable, and hence a natural inner bound on the true entanglement-assisted capacity region of a MAC. We expect that the latter is given by the regularized formula

$$\mathcal{C}_{\mathrm{ea},d}(N) = \overline{\bigcup_{n\in\mathbb{N}} \frac{1}{n}\mathcal{C}^{(1)}_{\mathrm{ea},d}(N^{\times n})}, \tag{10}$$

where $\overline{X}$ denotes the closure of a set X. For the developments in Section 4.3, we also define the *entanglement-assisted achievable rate region*

$$\mathcal{C}^{(1)}_{\mathrm{ea}}(N) \coloneqq \bigcup_{d\in\mathbb{N}} \mathcal{C}^{(1)}_{\mathrm{ea},d}(N), \tag{11}$$

which is achievable by the two senders sharing entanglement on quantum systems of arbitrarily large but finite local dimension.

Supplementary Note 2. Encoding a Non-local Game in a MAC

The following construction of a classical multiple access channel in terms of a non-local game is our main object of study. It is inspired by a similar construction of an interference channel (two senders, two receivers) in terms of the CHSH game in [4], and can also be found in unpublished work by Nötzel and Winter [5], a portion of which has appeared in [6]. Given a promise-free[3] non-local game $G = (\mathcal{X}_1, \mathcal{X}_2, \mathcal{Y}_1, \mathcal{Y}_2, W)$, we define the classical MAC $N_G\colon (\mathcal{X}_1 \times \mathcal{Y}_1) \times (\mathcal{X}_2 \times \mathcal{Y}_2) \to \mathcal{X}_1 \times \mathcal{X}_2$ as

$$N_G(\hat{x}_1, \hat{x}_2 | x_1, y_1; x_2, y_2) \coloneqq \begin{cases} \delta_{x_1\hat{x}_1}\delta_{x_2\hat{x}_2} & \text{if } (x_1, x_2, y_1, y_2) \in W, \\ (|\mathcal{X}_1||\mathcal{X}_2|)^{-1} & \text{else.} \end{cases} \tag{12}$$

In the above construction, to each player of the game we associate a sender in the MAC scenario with input alphabet $\mathcal{X}_i \times \mathcal{Y}_i$ for $i = 1, 2$. If the two senders input a question-answer tuple $(x_1, x_2, y_1, y_2) \in W$ that wins the non-local game G, the channel outputs the question pair (x_1, x_2); otherwise, the channel outputs a question pair drawn uniformly at random. In the following, for $i = 1, 2$ we denote by $X_i \sim \pi_{X_i}$ the random variables corresponding to the questions for Alice and Bob, by $Y_i \sim p_{Y_i|X_i}\pi_{X_i}$ the random variables corresponding to the answers, and by Z the random variable corresponding to the output of the channel N_G defined in (12) taking values in $\mathcal{X}_1 \times \mathcal{X}_2$.

As discussed in Section 1.2, the capacity region of a MAC is computed in terms of a product probability distribution $p_{X_1Y_1}(x_1, y_1)p_{X_2Y_2}(x_2, y_2)$ on the set of inputs to N. For the MAC (12), we can think of this input distribution in the following way: Given a product probability distribution $\pi(x_1, x_2) = \pi_{X_1}(x_1)\pi_{X_2}(x_2)$ on the question set, the players produce answers y_i to the game according to the probabilistic strategy $p_{Y_1|X_1}(y_1|x_1)p_{Y_2|X_2}(y_2|x_2)$ on which they agreed prior to starting the game. This allows us to connect the sum rate capacity of the channel N_G to the winning probability $\omega(G, \pi)$ as follows:

[3]Note that we can turn any game with promise into a promise-free one, as explained in Section 1.1.

Proposition 1. *Let $G = (\mathcal{X}_1, \mathcal{X}_2, \mathcal{Y}_1, \mathcal{Y}_2, W)$ be a promise-free non-local game, $\pi(x_1, x_2) = \pi_{X_1}(x_1)\pi_{X_2}(x_2)$ a probability distribution on the questions set $\mathcal{X}_1 \times \mathcal{X}_2$, and $p_{Y_1|X_1}(y_1|x_1)p_{Y_2|X_2}(y_2|x_2)$ a probabilistic strategy for Alice and Bob. For the MAC N_G defined in terms of G according to (12), let X_i, Y_i, Z be the random variables corresponding to the questions, answers, and channel output, respectively, as described above. We then have*

$$I(X_1Y_1X_2Y_2; Z) = H(Z) - p_L(\log|\mathcal{X}_1| + \log|\mathcal{X}_2|), \tag{13}$$

where $p_L = \sum_{(x_1,y_1,x_2,y_2)\notin W} \Pr(x_1, y_1, x_2, y_2)$ denotes the losing probability given the distribution $\pi(x_1, x_2)$ on the questions set and the probabilistic strategy $p_{Y_1|X_1}(y_1|x_1)p_{Y_2|X_2}(y_2|x_2)$.

Proof. We first expand the mutual information $I(X_1Y_1X_2Y_2; Z)$ as

$$I(X_1Y_1X_2Y_2; Z) = H(Z) - H(Z|X_1Y_1X_2Y_2). \tag{14}$$

Setting $d = |\mathcal{X}_1||\mathcal{X}_2|$ and recalling that $W \subset \mathcal{X}_1 \times \mathcal{Y}_1 \times \mathcal{X}_2 \times \mathcal{Y}_2$ is the winning set for G, the conditional entropy can be expressed as

$$\begin{aligned} H(Z|X_1Y_1X_2Y_2) &= \sum_{x_1,y_1,x_2,y_2} \Pr(x_1, y_1, x_2, y_2)H(Z|X_1 = x_1, Y_1 = y_1, X_2 = x_2, Y_2 = y_2) & (15)\\ &= \sum_{(x_1,y_1,x_2,y_2)\notin W} \Pr(x_1, y_1, x_2, y_2)H(Z|X_1 = x_1, Y_1 = y_1, X_2 = x_2, Y_2 = y_2) & (16)\\ &= \log d \sum_{(x_1,y_1,x_2,y_2)\notin W} \Pr(x_1, y_1, x_2, y_2) & (17)\\ &= p_L \log d, & (18) \end{aligned}$$

where the second equality follows since for $(x_1, y_1, x_2, y_2) \in W$ the channel N_G outputs (x_1, x_2) deterministically, and hence $H(Z|X_1 = x_1, Y_1 = y_1, X_2 = x_2, Y_2 = y_2) = 0$ in this case. □

Proposition 2. *Let $G = (\mathcal{X}_1, \mathcal{Y}_1, \mathcal{X}_2, \mathcal{Y}_2, W)$ be a promise-free non-local game with $\omega_U(G) < 1$, and consider the MAC N_G defined as in (12). Using the same notation as in Proposition 1, for all $0 < \delta < -\log\omega_U(G)$ there exists an $\varepsilon > 0$ such that*

$$I(X_1Y_1X_2Y_2; Z) \leq \max\{(1-\varepsilon)(\log|\mathcal{X}_1| + \log|\mathcal{X}_2|), \log|\mathcal{X}_1| + \log|\mathcal{X}_2| - \delta\}. \tag{19}$$

For a given $\delta > 0$ the maximal value of ε is given by the ε^* satisfying

$$\frac{\delta + h(\varepsilon^*)}{1 - \varepsilon^*} = \delta(\varepsilon^* \| 1 - \omega_U(G)), \tag{20}$$

where $h(x) = -x\log x - (1-x)\log(1-x)$ denotes the binary entropy and $\delta(x\|y) \coloneqq x\log\frac{x}{y} + (1-x)\log\frac{1-x}{1-y}$ denotes the binary relative entropy.

The strategy of the proof of Proposition 2 is the following: the goal of the proposition is to provide an upper bound on the sum rate capacity that separates it from the maximal value $\log|\mathcal{X}_1| + \log|\mathcal{X}_2|$. By the formula given in Proposition 1, the maximal value $\log|\mathcal{X}_1| + \log|\mathcal{X}_2|$ is attained if and only if p_L vanishes and $H(Z)$ attains its maximal value. We therefore need to show that we cannot have $p_L \approx 0$ and $H(Z) \approx \log|\mathcal{X}_1| + \log|\mathcal{X}_2|$ at the same time.

Proof of Proposition 2. We again set $d = |\mathcal{X}_1||\mathcal{X}_2|$. For the purpose of bounding the sum rate capacity $I(X_1Y_1X_2Y_2;Z)$ away from the maximal value $\log d$, we can assume without loss of generality that the losing probability $p_L = 1-\omega(G,\pi)$ for Alice and Bob is strictly positive, $p_L > 0$: In case $p_L = 0$, the probability distribution π on $\mathcal{X}_1 \times \mathcal{X}_2$ necessarily has support $\operatorname{supp}\pi$ strictly contained in $\mathcal{X}_1 \times \mathcal{X}_2$, as by assumption the game G cannot be won with certainty on receiving any one of the full set $\mathcal{X}_1 \times \mathcal{X}_2$ of questions. Hence,

$$I(X_1Y_1X_2Y_2;Z) \leq \log|\operatorname{supp}\pi| \leq \log(d-1) < \log d \tag{21}$$

in this case, as Alice and Bob have to lose on at least one question pair. Furthermore, we can assume w.l.o.g. that $p_L \leq 1-\omega_U(G)$, since $p_L > 1-\omega_U(G)$ and $\omega_U(G) < 1$ imply that

$$I(X_1Y_1X_2Y_2;Z) < \omega_U(G)\log d < \log d. \tag{22}$$

Therefore, we may assume that $0 < p_L \leq 1-\omega_U(G)$ for the remainder of the proof.

We prove the assertion of the theorem by contradiction. To this end, assume that

$$H(Z) \geq \log d - \delta \tag{23}$$

for some $0 < \delta < -\log\omega_U(G)$. Define a random variable W by

$$W = \begin{cases} 1 & \text{Alice and Bob win the game;} \\ 0 & \text{Alice and Bob lose the game,} \end{cases} \tag{24}$$

taking values 1 and 0 with probability $1-p_L$ and p_L, respectively. By the non-negativity of conditional entropy and (23) we have

$$H(W) + H(Z|W) = H(ZW) \geq H(Z) \geq \log d - \delta. \tag{25}$$

Expanding the left-hand side of (25) gives

$$h(p_L) + (1-p_L)H(X_1X_2) + p_L\log d \geq \log d - \delta, \tag{26}$$

which can be rearranged to

$$H(X_1X_2) \geq \log d - \gamma \tag{27}$$

with $\gamma := \frac{\delta + h(p_L)}{1-p_L}$. Observe that $D(\pi_{X_1}\pi_{X_2}\|\pi_U) = \log d - H(X_1X_2)$, and hence

$$\gamma \geq D(\pi_{X_1}\pi_{X_2}\|\pi_U). \tag{28}$$

Let now $Q = Q_{Y_1|X_1}Q_{Y_2|X_2}$ be the optimal probabilistic strategy for G given the distribution $\pi_{X_1}\pi_{X_2}$ on the questions, and denote by q_L the losing probability of the same strategy Q with questions drawn uniformly at random. Furthermore, let $\chi_W\colon \mathcal{X}_1 \times \mathcal{Y}_1 \times \mathcal{X}_2 \times \mathcal{Y}_2 \to \{0,1\}$ be the characteristic function of the winning set $W \subset \mathcal{X}_1 \times \mathcal{Y}_1 \times \mathcal{X}_2 \times \mathcal{Y}_2$. Applying the data-processing inequality with respect to $\chi_W \circ Q$ to (28), we obtain

$$\gamma \geq \delta(p_L\|q_L) \geq \delta(p_L\|1-\omega_U(G)), \tag{29}$$

where $\delta(x\|y) := x\log\frac{x}{y} + (1-x)\log\frac{1-x}{1-y}$ denotes the binary relative entropy, and the second inequality follows from the monotonicity of $y \mapsto \delta(x\|y)$ for $y \geq x$ and the fact that $q_L \geq 1-\omega_U(G)$.

The function $\gamma(x)=\frac{\delta+h(x)}{1-x}$ is monotonically increasing for all $x>0$, and $\lim_{x\to 0}\gamma(x)=\delta<-\log\omega_U(G)$ by assumption. On the other hand, $x\mapsto\delta(x\|1-\omega_U(G))$ is monotonically decreasing for $x\in[0,1-\omega_U(G)]$, and $\lim_{x\to 0}\delta(x\|1-\omega_U(G))=-\log\omega_U(G)$. Hence, there exists an $\varepsilon>0$ such that (29) is violated for all $p_L<\varepsilon$, which means that we either have $p_L\geq\varepsilon$ or the assumption (23) is false. In the first case,

$$I(X_1Y_1X_2Y_2;Z)=H(Z)-p_L\log d\leq(1-\varepsilon)\log d, \tag{30}$$

while in the second case,

$$I(X_1Y_1X_2Y_2;Z)<(1-p_L)\log d-\delta<\log d-\delta. \tag{31}$$

By the arguments above, for a given $\delta>0$ the maximal value of ε is given by the ε^* satisfying

$$\frac{\delta+h(\varepsilon^*)}{1-\varepsilon^*}=\delta(\varepsilon^*\|1-\omega_U(G)), \tag{32}$$

which concludes the proof. □

Remark 3. In applications of Proposition 2, the optimal (minimal) upper bound in Proposition 2 can be obtained by optimizing the right-hand side of (19) over $\delta\in(0,-\log\omega_U(G))$ and computing ε via (20).

Supplementary Note 3. Magic Square Game

Consider a 3×3-matrix whose rows and columns are labeled by $r,c\in\{0,1,2\}$, respectively. The magic square game G_{MS} [7–10] is a two-player game in which Alice and Bob receive questions $r,c\in\{0,1,2\}$ respectively, labeling a row r for Alice and a column c for Bob. They answer with 3-bit strings $s,t\in\{0,1\}^3$, where the bits in s,t correspond to the cells in r,c, respectively. Alice and Bob win the game if the following three conditions are satisfied:

1. the parity of Alice's bit string s is even: $s_0\oplus s_1\oplus s_2=0$;
2. the parity of Bob's bit string t is odd: $t_0\oplus t_1\oplus t_2=1$;
3. the bit strings agree in the overlapping cell (r,c): $s_c=t_r$.

3.1 Classical Strategies

The two parity constraints for Alice's and Bob's bit strings s and t render any deterministic perfect classical strategy for G_{MS} impossible, since the latter corresponds to a fixed valid filling of the nine cells of the magic square with bits such that conditions 1-3 above are satisfied. However, according to condition 1 the parity of all cells is even, while according to condition 2 this parity should be odd.

If the questions (r,c) are drawn uniformly at random, the best deterministic strategy for Alice and Bob consists in filling 8 of the 9 cells with valid bits. Hence, the optimal deterministic strategy has winning probability 8/9, and in fact $\omega_U(G_{\text{MS}})=8/9$ [10].

3.2 A Perfect Quantum Strategy

Brassard *et al.* [10] described the following perfect quantum strategy for the magic square game G_{MS} that is equivalent to the commuting observables strategy devised by Mermin [7] and Peres [8] depicted in Fig. 2 in the main text: Consider the 4-qubit entangled state

$$|\psi\rangle_{A_1A_2B_1B_2}=\frac{1}{2}\left(|00\rangle_{A_1A_2}|11\rangle_{B_1B_2}+|11\rangle_{A_1A_2}|00\rangle_{B_1B_2}-|01\rangle_{A_1A_2}|10\rangle_{B_1B_2}-|10\rangle_{A_1A_2}|01\rangle_{B_1B_2}\right), \tag{33}$$

where qubits A_1A_2 are with Alice, and B_1B_2 are with Bob. Furthermore, consider the following 2-qubit unitaries:

$$U_0 = \frac{1}{\sqrt{2}}\begin{pmatrix} \mathrm{i} & 0 & 0 & \mathrm{i} \\ 0 & -\mathrm{i} & 1 & 0 \\ 0 & \mathrm{i} & 1 & 0 \\ 1 & 0 & 0 & \mathrm{i} \end{pmatrix} \quad U_1 = \frac{1}{2}\begin{pmatrix} \mathrm{i} & 1 & 1 & \mathrm{i} \\ -\mathrm{i} & 1 & -1 & \mathrm{i} \\ \mathrm{i} & 1 & -1 & -\mathrm{i} \\ -\mathrm{i} & 1 & 1 & -\mathrm{i} \end{pmatrix} \quad U_2 = \frac{1}{2}\begin{pmatrix} -1 & -1 & -1 & 1 \\ 1 & 1 & -1 & 1 \\ 1 & -1 & 1 & 1 \\ 1 & -1 & -1 & -1 \end{pmatrix}$$
$$V_0 = \frac{1}{2}\begin{pmatrix} \mathrm{i} & -\mathrm{i} & 1 & 1 \\ -\mathrm{i} & -\mathrm{i} & 1 & -1 \\ 1 & 1 & -\mathrm{i} & \mathrm{i} \\ -\mathrm{i} & \mathrm{i} & 1 & 1 \end{pmatrix} \quad V_1 = \frac{1}{2}\begin{pmatrix} -1 & \mathrm{i} & 1 & \mathrm{i} \\ 1 & \mathrm{i} & 1 & -\mathrm{i} \\ 1 & -\mathrm{i} & 1 & \mathrm{i} \\ -1 & -\mathrm{i} & 1 & -\mathrm{i} \end{pmatrix} \quad V_2 = \frac{1}{\sqrt{2}}\begin{pmatrix} 1 & 0 & 0 & 1 \\ -1 & 0 & 0 & 1 \\ 0 & 1 & 1 & 0 \\ 0 & 1 & -1 & 0 \end{pmatrix} \tag{34}$$

Upon receiving the questions (r, c), Alice applies the unitary U_r to her qubits A_1A_2, while Bob applies V_c to his qubits B_1B_2. They each measure their respective qubits of the resulting state $U_r \otimes V_c|\psi\rangle$ in the computational basis and obtain measurement outcomes s_0s_1 and t_0t_1. As a last step, they complete their 2-bit outcome with a third bit such that the parity conditions of the magic square game are satisfied: Alice chooses s_2 such that $s_0 \oplus s_1 \oplus s_2 = 0$, while Bob chooses t_2 such that $t_0 \oplus t_1 \oplus t_2 = 1$. A lengthy but straightforward computation shows that this strategy indeed produces a valid answer pair (s, t) for every possible question pair (r, c).

3.3 MAC Based on the Magic Square Game

Specializing definition (12) to the magic square game G_{MS} described above, we set $\mathcal{R} = \{0, 1, 2\}$, $\mathcal{C} = \mathcal{R}$, $\mathcal{S} = \{0, 1\}^3$, $\mathcal{T} = \mathcal{S}$, and consider the following channel:

$$\begin{aligned} N_{G_{\mathrm{MS}}} &: (\mathcal{R} \times \mathcal{S}) \times (\mathcal{C} \times \mathcal{T}) \longrightarrow \mathcal{R} \times \mathcal{S} \\ N_{G_{\mathrm{MS}}}(\hat{r}, \hat{s}|r, s; c, t) &:= \begin{cases} \delta_{r\hat{r}}\delta_{s\hat{s}} & \text{if } (r, s; c, t) \in W, \\ \frac{1}{9} & \text{else,} \end{cases} \end{aligned} \tag{35}$$

where $W \subset \mathcal{R} \times \mathcal{S} \times \mathcal{C} \times \mathcal{T}$ is the subset of instances $(r, s; c, t)$ winning the magic square game.

Using the perfect quantum strategy for the magic square game detailed in Section 3.2, for any question pair (r, c) Alice and Bob can produce answers (s, t) such that $(r, s, c, t) \in W$. Hence, with a uniform distribution over the questions $\mathcal{R} \times \mathcal{C}$ they can achieve the maximal sum rate of $\log 9 \approx 3.16993$ for the magic-square-MAC (35). To bound the sum rate achievable by classical strategies corresponding to product input distributions on $(\mathcal{R} \times \mathcal{S}) \times (\mathcal{C} \times \mathcal{T})$, our goal is to find the smallest upper bound on $I(RSCT; Z)$ given by Proposition 2 (we again use capital Latin letters for the random variables corresponding to the question and answer sets, as well as Z for the channel output random variable):

$$I(RSCT; Z) \leq \max\{(1 - \varepsilon^*) \log 9, \log 9 - \delta\} =: u(\delta) \tag{36}$$

for some $\delta \in (0, \log \frac{9}{8})$ and the corresponding optimal ε^* determined through (20). As explained in Remark 3, we find the optimal $\delta^* = 0.03299$ (using, e.g., Mathematica), which yields $\varepsilon(\delta^*) = 0.01040$ and $I(RSCT; Z) \leq u(\delta^*) = 3.13694$. In Supplementary Fig. 3 we plot the upper bound (36) as a function of $\delta \in [0, \log 9/8]$.

We can compare the upper bound $u(\delta^*) = 3.13694$ to a lower bound on the sum rate computed by numerically maximizing the mutual information $I(RSCT; Z)$ with respect to product probability distributions (see (2)). Carrying out this optimization in MATLAB in repeated runs using different random starting points gives a lower bound of 2.84195 on the true maximum. Assuming that this value is close to the true

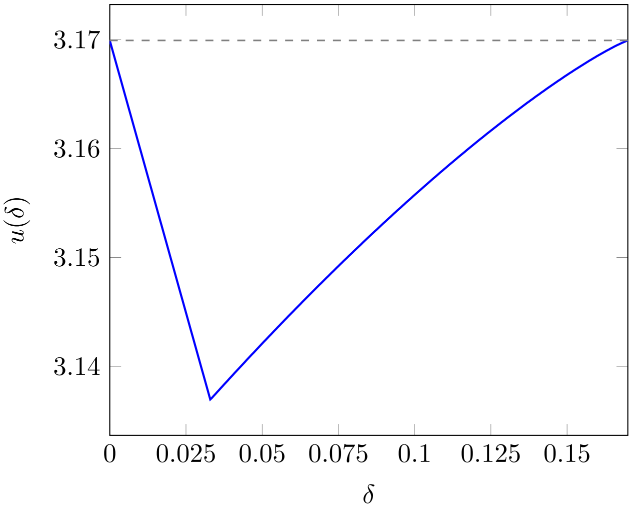

Supplementary Fig. 3: Upper bounds on the classical sum rate for the multiple access channel based on the magic square game.
Plotted is the upper bound $u(\delta)$ defined in (36) as a function of δ, with $\varepsilon = \varepsilon(\delta)$ chosen maximally such that (29) is violated. The minimum occurs at $\delta^* = 0.03299$ giving $\varepsilon(\delta^*) = 0.01040$ and $u(\delta^*) = 3.13694$.

maximum, this result suggests that our upper bound $u(\delta^*) = 3.13694$ on the sum rate can likely be further improved. We also computed an inner bound on the capacity region $\mathcal{C}$ of the MAC (35) using the method detailed in [11, Sec. II.A]. This inner bound on the capacity region is plotted in Supplementary Fig. 4.

We briefly comment on a different type of entanglement assistance for a MAC where each sender shares entanglement with the receiver. This communication scenario was discussed by Hsieh *et al.* [12] for quantum multiple access channels $\mathcal{N}\colon A'B' \to C$ mapping quantum systems A' in Alice's possession and B' in Bob's possession to a quantum system C in possession of the receiver Charlie. In addition to entanglement assistance, quantum MACs have been studied in various other scenarios [13–17].

The following capacity region for entanglement-assisted quantum MACs is proved in [12]: Let $|\phi\rangle_{AA'}$ and $|\psi\rangle_{BB'}$ be pure quantum states, and set $\omega_{ABC} = (\mathrm{id}_A \otimes \mathrm{id}_B \otimes \mathcal{N}_{A'B'\to C})(\phi_{AA'} \otimes \psi_{BB'})$. Let $\mathcal{C}_E(\mathcal{N}, \phi, \psi)$ be the set of all non-negative rate pairs (R_1, R_2) satisfying

$$R_1 \leq I(A; C|B) \tag{37}$$
$$R_2 \leq I(B; C|A) \tag{38}$$
$$R_1 + R_2 \leq I(AB; C), \tag{39}$$

where the quantum (conditional) mutual informations on the right-hand sides are evaluated on the state ω_{ABC}. Define $\widetilde{\mathcal{C}}_E(\mathcal{N})$ as the union over all states ϕ and ψ. Then the entanglement-assisted capacity region $\mathcal{C}_E(\mathcal{N})$ of a quantum MAC $\mathcal{N}$ is equal to

$$\mathcal{C}_E(\mathcal{N}) = \overline{\bigcup_{n\in\mathbb{N}} \frac{1}{n} \widetilde{\mathcal{C}}_E(\mathcal{N}^{\otimes n})}. \tag{40}$$

Moreover, we have the following single-letter upper bound on the sum rate:

$$R_1 + R_2 \leq \max_{\phi_{AA'}, \psi_{BB'}} I(AB; C). \tag{41}$$

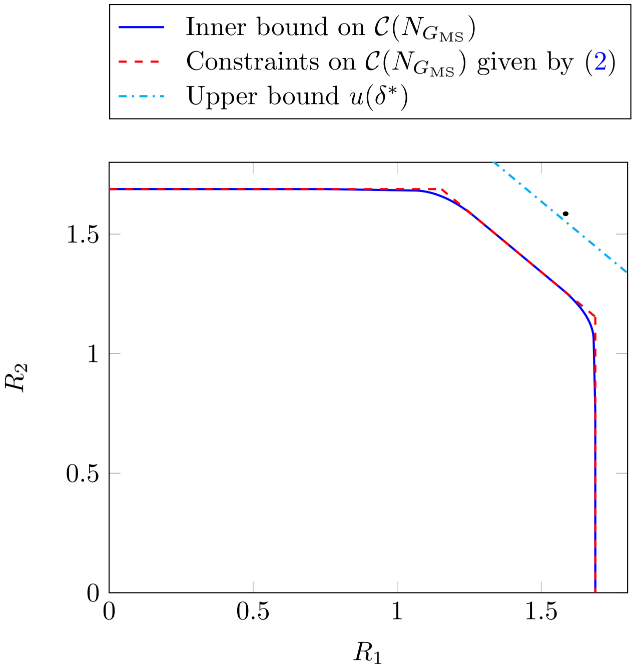

Supplementary Fig. 4: Inner and outer bounds on the capacity region of the multiple access channel based on the magic square game.
The inner bound on the capacity region $\mathcal{C}(N_{G_{\mathrm{MS}}})$ of the MAC (35) based on the magic square game is shown in solid blue. Approximate values of the outer pentagonal bound on $\mathcal{C}$ given by optimizing the individual constraints in (2) for R_1, R_2 and $R_1 + R_2$ are marked by dashed red lines. The dash-dotted cyan line is the (optimized) upper bound on the sum rate from Proposition 2. The black dot is the rate pair $(\log 3, \log 3)$ achievable by the entanglement-assisted coding strategy explained in Section 3.2.

We now specialize the above entanglement-assisted setting to classical MACs $N\colon \mathcal{A}\times\mathcal{B} \to \mathcal{Z}$ as introduced in Section 1.2. Any classical channel necessarily completely dephases a quantum system with respect to some fixed basis. Hence, choosing bases $\{|i\rangle_A\}$ and $\{|i\rangle_B\}$ and fixing pure quantum states $\phi_{AA'}$ and $\psi_{BB'}$, the joint input state of Alice and Bob for a classical MAC is of the form

$$\sum_{i,j} p_i p_j \phi_A^i \otimes |i\rangle\langle i|_{A'} \otimes \psi_B^j \otimes |j\rangle\langle j|_{B'}, \tag{42}$$

where $\{p_i\}$ with $p_i = \mathrm{tr}(|i\rangle\langle i|_{A'}\phi_{AA'})$ and $\{p_j\}$ with $p_j = \mathrm{tr}(|j\rangle\langle j|_{B'}\psi_{BB'})$ are probability distributions, and $\phi_A^i = \frac{1}{p_i}\langle i|\phi|i\rangle_{A'}$ and $\phi_A^j = \frac{1}{p_j}\langle j|\psi|j\rangle_{B'}$. The MAC N maps the joint input state in (42) to a state

$$\omega_{ABZ} = \sum_{i,j} p_i p_j N(k|i,j) \phi_A^i \otimes \psi_B^j \otimes |k\rangle\langle k|_Z. \tag{43}$$

On the other hand, consider a classical state

$$\theta_{ABZ} = \sum_{i,j} p_i p_j N(k|i,j) |i\rangle\langle i|_A \otimes |j\rangle\langle j|_B \otimes |k\rangle\langle k|_Z, \tag{44}$$

and observe that ω_{ABZ} can be obtained from θ_{ABZ} by a quantum operation that first measures the (classical) systems AB in θ and depending on the outcome (i,j) prepares the state $\phi_A^i \otimes \psi_B^j$. Hence, by the data

processing inequality for the quantum mutual information we have

$$I(AB;Z)_\omega \le I(AB;Z)_\theta, \tag{45}$$

and $I(AB;Z)_\theta$ is the classical mutual information with respect to the product probability distribution $p_i p_j$ appearing in the sum rate constraint for the classical MAC N given in (2).

From the above discussion and (41), we conclude that for a classical MAC entanglement shared between each sender and the receiver cannot increase the achievable sum rate $R_1 + R_2$. In contrast, we showed in this section that entanglement shared between the senders can indeed increase the sum rate up to the maximal value.

Supplementary Note 4. Linear System Games

In this section we discuss non-local games G_{LS} based on linear systems of equations [18]. Let $Ax = b$ be an $m \times n$ linear system of equations over $\mathbb{F}_2$. We denote by $V_i = \{j \in [n]\colon A_{ij} \neq 0\}$ the indices of variables appearing the i-th equation of the linear system. In the linear system game, Alice receives as a question an index $i \in [m]$ labeling a row in the linear system. She replies with a vector $y \in \mathbb{F}_2^n$ of values for x such that $\sum_{j\in V_i} y_j = b_i$. Bob receives as a question an index $j \in [n]$, and he answers with a bit x_j corresponding to an assignment of the variable x_j. Alice and Bob win the game if either $j \notin V_i$ or $y_j = x_j$.

A linear system game G_{LS} defined in terms of a linear system $Ax = m$ can be associated with a certain finitely-presented group $\Gamma(A,b)$ called a solution group. The maximal winning probability using quantum strategies can then be related to approximate representations of $\Gamma(A,b)$ [19]. Slofstra & Vidick [20] showed that suitable approximate representations of $\Gamma(A,b)$ (giving rise to near-perfect quantum strategies) do exist provided the dimension of the representation space, called the hyperlinear profile, is large enough. They exhibited a particular example G_{SV} of a linear system game based on a suitable solution group $\Gamma(A,b)$, for which the above observations can be translated into lower and upper bounds on the local dimension d of any quantum strategy for G_{SV}. In terms of the losing probability $p_L = 1 - \omega_U(G_{\mathrm{SV}})$ and constants C, C', the following bounds are proved in [20]:

$$\frac{C}{p_L^{1/6}} \le d \le \frac{C'}{p_L^{1/2}}. \tag{46}$$

4.1 Limiting the Entanglement Assistance

Proposition 4. *If Alice and Bob are constrained to quantum strategies with dimension at most d, then the sum rate capacity of $N_{G_{\mathrm{SV}}}$ is bounded away from perfect, i.e., $\log m + \log n$, by $\Theta(\frac{1}{d^{13}})$.*

Proof. Let G_{SV} be the linear system game defined in [20]. By the discussion above and (46), we have the following lower bound for the losing probability if Alice and Bob only use d-dimensional quantum strategies:

$$1 - \omega_U(G_{\mathrm{SV}}) \ge \frac{C_1}{d^6}, \tag{47}$$

for some constant $C_1 > 0$. In order to use (2), we let $\delta = \frac{C_1}{d^{13}}$ and assume that $\varepsilon^* < \delta$. For large d, we can upper-bound the left-hand side of eq. (20) by

$$\frac{\delta + h(\delta)}{1-\delta} \ge \delta(\varepsilon^* \| 1 - \omega(G_{\mathrm{SV}})), \tag{48}$$

where we used $h(\varepsilon^*) \le h(\delta)$ whenever $\delta < \frac{1}{2}$. Next, observe that for $\delta \in [0, \frac{1}{2}]$ the binary entropy term $h(\delta)$ is upper-bounded by $a\delta^\alpha$ for $\alpha < 1$ and a large enough. Letting $\alpha = \frac{25}{26}$, we can underestimate the right-hand side via Pinsker's inequality and get

$$\delta(\varepsilon^* \| 1 - \omega_U(G_{\mathrm{SV}})) \ge \frac{2}{\ln 2}\Big[\varepsilon^* - (1 - \omega_U(G_{\mathrm{SV}}))\Big]^2 \ge \frac{2}{\ln 2}\Big[\varepsilon^* - \frac{C_1}{d^6}\Big]^2 \ge \frac{2}{\ln 2}\Big[\frac{C_1^2}{d^{12}} - 2\delta\frac{C_1}{d^6}\Big] \tag{49}$$

Putting it all together, we conclude the following inequality

$$\frac{\delta + a\delta^\alpha}{1-\delta} \ge \frac{2C_1^2}{\ln 2}\Big[\frac{1}{d^{12}} - 2\frac{1}{d^{19}}\Big]. \tag{50}$$

Observe that as d goes to infinity, the right-hand side goes as $1/d^{12}$, while the left-hand side goes as $1/d^{12.5}$. At some large enough d, (50) is violated. ε^* cannot be smaller than δ for large enough d. Hence, by (2) we have the following upper bound on the sum rate capacity:

$$I(X_1Y_1X_2Y_2; Z) \le \log n + \log m - \frac{C_1}{d^{13}} \tag{51}$$

Since d-dimensional quantum strategies subsume all lower dimensional strategies, this converse provides a limit, if implicit, on how well $N_{G_{\mathrm{SV}}}$ can be used for strategies with small dimension. □

4.2 Achievable Strategies Using d-dimensional Maximally Entangled States

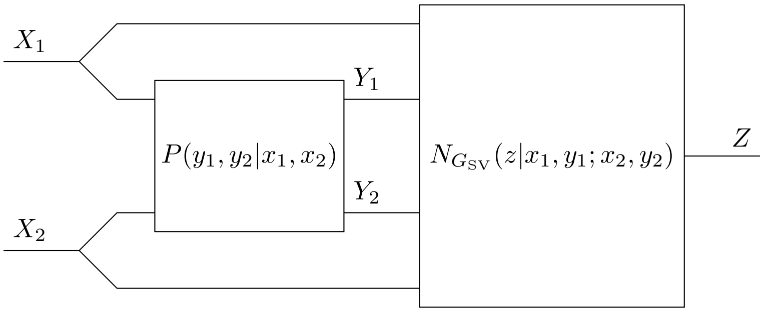

Supplementary Fig. 5: Entanglement-assisted coding strategy for the multiple access channel based on a linear system game.
The MAC $N_{G_{\mathrm{SV}}}$ is defined in terms of the linear system game G_{SV} discussed in Section 4. The correlation P produced by the quantum strategy is detailed in [20].

In this section we prove the existence of a sequence of coding strategies for the MAC $N_{G_{\mathrm{SV}}}$ defined in terms of the $m \times n$-linear system game G_{SV} described above that achieves the rate pair $(\log m, \log n)$ in the achievable rate region in the limit $d \to \infty$.

Proposition 5. *Let G_{SV} be the linear system game from [20] associated with the $m \times n$-linear system $Ax = b$, and let $N_{G_{\mathrm{SV}}}$ be the MAC defined in terms of G_{SV} via (12). Assume that the two players share a maximally entangled state $|\psi\rangle \in \mathbb{C}^d \otimes \mathbb{C}^d$ of Schmidt rank d sufficiently large. Then there is a coding strategy that achieves the rate pair $R = (R_1, R_2)$, where*

$$R_1 = (1-p_L)\log m - (1-p_L)(f(d)\log(nm-1) + h(f(d))) - \frac{p_L}{2}\log(nm-1) - h(p_L) \tag{52}$$

$$R_2 = (1-p_L)\log n - (1-p_L)(f(d)\log(nm-1) + h(f(d))) - \frac{p_L}{2}\log(nm-1) - h(p_L). \tag{53}$$

For this coding strategy, both the losing probability p_L and the function $f(d)$ vanish in the limit $d \to \infty$.

Proof. In order to prove the claim of the proposition, we make use of the following easily-verifiable entropic inequalities:

$$I(A;B) - I(A;B|D) = -H(A|B) + I(A;D) + H(A|BD) \geq -H(A|B) \tag{54}$$

$$I(A;B|C) - I(A;B|CD) = I(A;D|C) + H(D|ABC) - H(D|BC) \geq -H(D|BC) \tag{55}$$

$$I(A;B|CD) - I(A;BC|D) = -I(A;C|D) \tag{56}$$

Let X_i, Y_i be the random variables associated to the questions and answers for players $i = 1, 2$, let Z be the random variable associated to the output of the MAC $N_{G_{\mathrm{SV}}}$, and let W be the random variable indicating a win defined in (24). We fix the following coding strategy: Alice and Bob draw the questions x_1 and x_2 uniformly at random, and produce y_1 and y_2 using the quantum strategy detailed in [20] based on measuring a maximally entangled state ψ, as depicted in Supplementary Fig. 5. In terms of the general entanglement-assisted coding scenario described in Section 1.3, this corresponds to setting $A_i = X_i$, $X'_i = Y_i$, and using the trivial post-processing $f_i(x_j, y_j|x_i, y_i) = \delta_{x_i,x_j}\delta_{y_i,y_j}$. By the right-hand inequality in (46) (which is proved in Theorem 1.1 in [20]), the above strategy has losing probability

$$p_L \leq \left(\frac{C'}{d}\right)^2 \tag{57}$$

for some constant C'.

We first determine an achievable rate R_1 for the first sender satisfying $R_1 \leq I(Z;X_1|X_2)$ (see (2) and the discussion in Section 1.3). To this end, we use (55) with the choices $A = Z$, $B = X_1$, $C = X_2$, $D = W$ to obtain

$$\begin{aligned}
I(Z;X_1|X_2) &\geq I(Z;X_1|X_2W) - H(W|X_1X_2) && (58)\\
&= I(Z;X_1X_2|W) - I(Z;X_2|W) - H(W|X_1X_2) && (59)\\
&\geq I(Z;X_1X_2|W) - I(Z;X_2|W) - h(p_L) && (60)\\
&= (1-p_L)\left[H(X_1X_2|W=1) - H(X_2|W=1)\right] - h(p_L) && (61)\\
&\geq (1-p_L)\left[H(X_1X_2|W=1) - \log n\right] - \frac{p_L}{2}\log(nm-1) - h(p_L). && (62)
\end{aligned}$$

In the second line we used (56) and in the third line we used $H(W|X_1X_2) \leq H(W) \leq h(p_L)$. In the fourth line we used that, if Alice and Bob win the game ($W = 1$), then the variable Z is a deterministic function of X_1X_2 and hence $I(Z;X_1Y_1X_2Y_2|W = 1) = H(X_1X_2|W = 1)$, together with the fact that $I(Z;X_1Y_1X_2Y_2|W = 0) = 0$. Finally, in the last line we used the trivial bound $H(X_2|W = 1) \leq \log|\mathcal{X}_2| = n$ as well as the fact that $\frac{p_L}{2}\log(nm-1) \geq 0$.

We now bound the entropy $H(X_1X_2|W = 1)$ in (62) by considering the probability distribution

$$\pi^W_{X_1X_2} = \{\Pr(X_1X_2 = x_1x_2|W = 1)\}_{x_1,x_2}. \tag{63}$$

Our goal is to show that $\pi^W_{X_1X_2}$ converges to the uniform distribution π_U on $\mathcal{X}_1 \times \mathcal{X}_2$ in total variation distance as $d \to \infty$. By continuity of entropy, this then implies that $H(X_1X_2|W = 1) \approx \log m + \log n$ with the approximation error vanishing in the limit $d \to \infty$.

To show this claim, we use Bayes' theorem to express $\Pr(X_1X_2 = x_1x_2|W = 1)$ as

$$\begin{aligned}
\Pr(X_1X_2 = x_1x_2|W=1) &= \frac{\Pr(W=1|X_1X_2 = x_1x_2)\Pr(X_1X_2 = x_1x_2)}{\Pr(W=1)} && (64)\\
&= \frac{1}{nm}\frac{\Pr(W=1|X_1X_2 = x_1x_2)}{\Pr(W=1)}. && (65)
\end{aligned}$$

Due to (57), the winning probability satisfies

$$\Pr(W=1) = 1 - p_L \geq 1 - (C'/d)^2. \tag{66}$$

Moreover, by Lemma 4.2 in [20] every strategy that achieves a winning probability of at least $1-p_L$ wins with probability $1-nmp_L$ on any question (x_1, x_2), and hence

$$\Pr(W=1|X_1X_2 = x_1x_2) \geq 1 - nmp_L \geq 1 - nm(C'/d)^2. \tag{67}$$

For the total variation distance $d_{\mathrm{TV}}(\pi^W_{X_1X_2}, \pi_U)$, the bounds (66) and (67) imply that

$$d_{\mathrm{TV}}(\pi^W_{X_1X_2}, \pi_U) = \frac{1}{2}\sum_{x_1,x_2}\left|\Pr(X_1X_2 = x_1x_2|W=1) - \frac{1}{nm}\right| =: f(d) \tag{68}$$

for some non-negative function $f(d)$ that converges to zero as $d \to \infty$. By the continuity of entropy [21],

$$H(X_1X_2|W=1) \geq \log m + \log n - f(d)\log(nm-1) - h(f(d)), \tag{69}$$

and substituting this in (62) yields $R_1 \leq I(Z; X_1|X_2)$ with

$$R_1 \coloneqq (1-p_L)\log m - (1-p_L)(f(d)\log(nm-1) + h(f(d))) - \frac{p_L}{2}\log(nm-1) - h(p_L). \tag{70}$$

Using similar steps as above, we can also show that $R_2 \leq I(Z; X_2|X_1)$ with

$$R_2 \coloneqq (1-p_L)\log n - (1-p_L)(f(d)\log(nm-1) + h(f(d))) - \frac{p_L}{2}\log(nm-1) - h(p_L). \tag{71}$$

For the rate pair (R_1, R_2) to be achievable, it remains to be shown that $R_1 + R_2$ satisfies the sum rate constraint $R_1 + R_2 \leq I(Z; X_1X_2)$. To this end, we use (54) with the choices $A = Z$, $B = X_1X_2$, $D = W$ to obtain

$$\begin{aligned} I(Z; X_1X_2) &\geq I(Z; X_1X_2|W) - H(Z|X_1X_2) && (72)\\ &= (1-p_L)H(X_1X_2|W=1) - H(Z|X_1X_2) && (73)\\ &\geq (1-p_L)(\log m + \log n) - (1-p_L)(f(d)\log(nm-1) + h(f(d))) - H(Z|X_1X_2), && (74) \end{aligned}$$

which follows from the discussion above and (69). To bound the conditional entropy $H(Z|X_1X_2)$, note that $\Pr(Z \neq X_1X_2) = p_L\frac{nm-1}{nm} \leq p_L$, and hence we can apply Fano's inequality to obtain the bound

$$H(Z|X_1X_2) \leq p_L\log(nm-1) + h(p_L). \tag{75}$$

Substituting this in (74) yields

$$\begin{aligned} I(Z; X_1X_2) &\geq (1-p_L)(\log m + \log n) - (1-p_L)(f(d)\log(nm-1) + h(f(d))) - p_L\log(nm-1) - h(p_L) && (76)\\ &\geq (1-p_L)(\log m + \log n) - 2(1-p_L)(f(d)\log(nm-1) + h(f(d))) - p_L\log(nm-1) - 2h(p_L) && (77)\\ &= R_1 + R_2 && (78) \end{aligned}$$

with R_1 and R_2 as in (70) and (71), respectively. This finishes the proof. □

By Proposition 5, the achievable rate region $\mathcal{C}^{(1)}_{\mathrm{ea},d}(N_{G_{\mathrm{SV}}})$ gets arbitrarily close to the rate pair $(\log m, \log n)$ in the limit $d \to \infty$. Hence, we have the following result:

Corollary 6. *Let G_{SV} be the linear system game from [20] associated with the $m \times n$-linear system $Ax = b$, and let $N_{G_{\mathrm{SV}}}$ be the MAC defined in terms of G_{SV} via (12). Then the rate pair $(\log m, \log n)$ is contained in the closure of $\mathcal{C}^{(1)}_{\mathrm{ea}}(N_{G_{\mathrm{SV}}})$.*

4.3 Undecidability of the Rate Region of a MAC

Propositions 4 and 5 show that there is a MAC $N_{G_{\mathrm{SV}}}$ defined in terms of the $m \times n$-linear system game G_{SV} such that the sum rate capacity $S_{\mathrm{ea},d}(N_{G_{\mathrm{SV}}})$ is bounded away from $(\log m, \log n)$ for any finite d, but the boundary of the d-entanglement-assisted single-letter capacity region $\mathcal{C}^{(1)}_{\mathrm{ea},d}(N_{G_{\mathrm{SV}}})$ gets arbitrarily close to $(\log m, \log n)$ in the limit $d \to \infty$.

Using a recent result by Slofstra [19], we can even prove the following: for a general linear system game G_{LS} and the corresponding MAC $N_{G_{\mathrm{LS}}}$, it is undecidable to determine if the maximal rate $(\log m, \log n)$ is achievable with finite-dimensional entanglement assistance:

Proposition 7. *Let $N_{G_{\mathrm{LS}}}$ be the MAC defined via* (12) *in terms of an $m \times n$-linear system game G_{LS}. Then it is undecidable to determine if the rate pair $(\log m, \log n)$ belongs to $\mathcal{C}^{(1)}_{\mathrm{ea}}(N_{G_{\mathrm{LS}}})$.*

Proof. Let G_{LS} be the game associated to the $m \times n$-linear system $Ax = b$. Then Corollary 1.3 in [19] proves that it is undecidable to determine if G_{LS} has a perfect strategy in the set of finite-dimensional quantum correlations as defined in (6). If there is a perfect strategy, then by the construction of $N_{G_{\mathrm{LS}}}$ the two senders can code at the rate pair $(\log m, \log n)$ by drawing the questions x_i uniformly at random and using the perfect strategy to produce y_i such that $(x_1, y_1, x_2, y_2) \in W$. Conversely, if there is no perfect strategy and hence $\omega_U(G_{\mathrm{LS}}) < 1$, then for any finite d the sum rate capacity $S_{\mathrm{ea},d}(N_{G_{\mathrm{LS}}})$ can be bounded away from $\log m + \log n$ using Proposition 2, and this separates the point $(\log m, \log n)$ from the entanglement-assisted rate-region $\mathcal{C}^{(1)}_{\mathrm{ea}}(N_{G_{\mathrm{LS}}})$. Hence, the pair $(\log m, \log n)$ belongs to $\mathcal{C}^{(1)}_{\mathrm{ea}}(N_{G_{\mathrm{LS}}})$ if and only if there is a perfect strategy for G_{LS}, which is undecidable. □

Supplementary Note 5. Hardness of Computing the Capacity Region of MACs

Despite the availability of a single-letter characterization, as given by (2), computing the capacity region of an arbitrary multiple access channel is a difficult task [22]. The difficulty lies in the inherent non-convexity of the problem, i.e., the optimization is constrained to be over product distributions [11]. In this section, we show that deciding if a MAC can be used perfectly or not (up to $\Theta(\frac{1}{n^3})$) is NP-hard. This implies that deciding if an arbitrary point (R_1, R_2) belongs to the capacity region to within an additive error of $\Theta(\frac{1}{n^3})$ is NP-hard.

5.1 The PCP Theorem

The results to follow rely on the probabilistically checkable proofs (PCP) theorem, which says that any language in the class NP admits a characterization via probabilistically checkable proofs [23–25]. More formally, let $\mathrm{PCP}_{c,s}[r(n), q(n)]$ be the class of all languages L such that there exists a verifier V, which is free to use $\mathcal{O}(r(n))$ random bits and query a given proof $\mathcal{O}(q(n))$ times, with the following properties:

1. Completeness: If $x \in L$, then there exists a proof P such that V accepts with probability at least c.
2. Soundness: If $x \notin L$, then V accepts with probability at most s.

Note that this can be considered a generalization of NP as $\mathrm{NP} = \mathrm{PCP}_{1,0}[0, \mathrm{poly}(n)]$. The original PCP theorem says that $\mathrm{NP} \subseteq \mathrm{PCP}_{1,1/2}[\log n, 1]$ [26]. To illustrate its implications, consider the canonical NP-complete language 3SAT for example. Take a Boolean formula ψ in 3-conjunctive normal form (3CNF), i.e., it is a conjunction of clauses that are disjunctions of three literals. Note that a literal can be a Boolean

variable or its negation. Say $\psi \notin$ 3SAT. A verifier exists such that, with access to logarithmic randomness and a constant number of queries to a given proof or witness, it will reject with non-trivially high probability. This suggests that proving a falsehood, e.g., $\psi \in$ 3SAT when that is not the case, typically involves making many errors.

The PCP theorem can be equivalently formulated as a statement about the hardness of approximating NP-complete problems [27, 28]. We will restrict our attention to the following formulation.

Theorem 8 (PCP theorem; [29, 30])**.** *Given a 3-CNF-5 Boolean formula ψ, to decide whether ψ has a satisfying assignment or that every assignment violates at least $(1-c)$ fraction of the clauses in ψ is NP-hard, for some constant $c < 1$.*

Here, a formula ψ is called 3-CNF-5 if it is a conjunction of m clauses and each clause is a disjunction of exactly three distinct literals and each of the n Boolean variables appears in exactly five clauses. Remark that the number of clauses m is $\mathcal{O}(n)$. We call ψ at most c-satisfiable for some $c \in [0,1]$ if some assignment satisfies f fraction of its clauses, for $f \in [0,c]$, and no assignments satisfies more than c fraction of its clauses.

5.2 The Basic Two-Prover Game

We denote by G_{H} the non-local game version of the basic two-prover protocol introduced in [28]. Namely, given a 3-CNF-5 Boolean formula $\psi = C_1 \wedge C_2 \wedge ... \wedge C_m$ as input, where $C_j = y_{a_j} \vee y_{b_j} \vee y_{c_j}$, the referee does the following:

1. Choose an integer $j \in \{1,...,m\}$ uniformly at random and send j to Alice. Choose $k \in \{a_j, b_j, c_j\}$ uniformly at random and send k to Bob.

2. Receive an assignment for C_j from Alice and a truth value for x_k from Bob. They win if Alice's answer satisfies C_j and the two agree on the value of x_k, otherwise they lose.

Let ψ be at most c-satisfiable. Because the optimal strategy is deterministic, Bob will have an assignment to ψ. If the clause in the question to Alice is violated by Bob's assignment, then the best Alice can do is disagree with Bob on the value of one Boolean variable in the clause and hope that Bob did not receive it as a question. This implies that $\omega(G_{\mathrm{H}}) \leq \frac{2+c}{3}$. Conversely, $\omega(G_{\mathrm{H}}) \leq \frac{2+c}{3}$ implies that ψ is at most c-satisfiable. To see this, note that if some assignment satisfies more than c fraction of the clauses in ψ, then Alice and Bob can use it to win with probability higher than $\frac{2+c}{3}$. Using the PCP theorem, these observations, in addition to the fact that $\psi \in 3SAT \Leftrightarrow \omega(G_{\mathrm{H}}) = 1$, imply that it is NP-hard to decide if G_{H} can be won with probability one or with probability at most $\frac{2+c}{3}$.

5.3 Hardness Result

If the game is made promise-free, then it follows that it is NP-hard to decide if $\omega_U(G_{\mathrm{H}}) = 1$ or $\omega_U(G_{\mathrm{H}}) \leq 1 - (\frac{1-c}{n})$.

Proposition 9. *It is NP-hard to decide if the sum capacity of the MAC associated with the promise-free version of G_{H} is equal to its maximum value $\log m + \log n$ or it is bounded away from it by $\Theta(\frac{1}{n^3})$.*

Proof of Proposition 9. Observe that if ψ has a satisfying assignment, then the two senders can use the channel perfectly, i.e., $R_1 = \log m$ and $R_2 = \log n$. On the other hand, if ψ has no satisfying assignment, then $\omega_U(G_{\mathrm{H}})$ is strictly less than 1. Hence, we can use Proposition 2 to make statements about the sum

capacity in a manner similar to (4). Let $\delta = \frac{(1-c)}{n^3}$ and assume that $\varepsilon^* < \delta$. For large n, we can overestimate the left-hand side of eq. (20) by

$$\frac{\delta + b\delta^\beta}{1-\delta} \geq \delta(\varepsilon^* \| 1 - \omega_U(G_{\mathrm{H}})), \tag{79}$$

where $\beta = \frac{5}{6}$ and b is taken to be large enough. Again, we use Pinsker's inequality to lower bound the right hand side.

$$\frac{\delta + b\delta^\beta}{1-\delta} \geq \frac{2(1-c)^2}{\ln 2}\left[\frac{1}{n^2} - 2\frac{1}{n^4}\right] \tag{80}$$

As n goes to infinity, the right-hand side goes as $1/n^2$, while the left-hand side goes as $1/n^{2.5}$. ε^* cannot be smaller than δ for large enough n. Therefore, whenever $\omega_U(G_{\mathrm{H}}) < 1$, i.e., ψ has no satisfying assignment, we conclude from eq. (19) that for all large enough n,

$$I(X_1Y_1X_2Y_2; Z) \leq \log m + \log n - \frac{(1-c)}{n^3} \tag{81}$$

The proposition follows from here via the PCP theorem. □

It is instructive to compare this hardness result with the time complexity of the popular Arimoto-Blahut (AB) algorithm for computing the point-to-point discrete channel capacity [31, 32]. If we consider the two senders together, then the channel capacity is the solution to a convex program. The number of iterations needed in order to have $\frac{\epsilon}{n^3}$ additive precision for the capacity using the AB algorithm is $\frac{\mathcal{O}(n^3 \log n)}{\epsilon}$ in the worst case. Assuming P $\neq$ NP, there is no polynomial-time algorithm to get to within the same precision for the boundary of the capacity region of an arbitrary discrete MAC. Moreover, assuming the exponential time hypothesis [33], there is no sub-exponential algorithm to compute the boundary of the region to inverse cubic precision. In such a case, one may consider the "naive" method of covering the space of product probability distributions with a net and computing an approximation of the capacity region. We argue below that, assuming the validity of the exponential time hypothesis, this net covering method is not far from optimal.

Let $K \subseteq \mathbb{R}^n$ be a subset of the Euclidean space $\mathbb{R}^n$ and $\varepsilon > 0$. An *ε-net* for K is a subset $N \subseteq K$ such that every point of K is within distance ε of a net point in N. We denote by $C(K,\varepsilon)$ the *covering number* of K, defined as the smallest possible cardinality of an ε-net N for K. By a standard volume argument, $C(K,\varepsilon)$ is bounded from below as

$$C(K,\varepsilon) \geq \frac{|K|}{|\mathcal{B}_\varepsilon^n|}, \tag{82}$$

where $|K|$ denotes the (Euclidean) volume of K embedded in $\mathbb{R}^n$, and $\mathcal{B}_\varepsilon^n$ is the n-ball with radius ε. Let now $K = \Delta_n$ be the n-probability simplex, and recall that

$$|\Delta_n| = \frac{\sqrt{n}}{(n-1)!} \qquad \text{and} \qquad |\mathcal{B}_\varepsilon^n| = \frac{\pi^{n/2}}{\Gamma(\frac{n}{2}+1)}\varepsilon^n. \tag{83}$$

Here, $\Gamma(\cdot)$ is the well-known Gamma function, satisfying $\Gamma(n) = (n-1)!$ for $n \in \mathbb{N}$. Using the Stirling approximation $n! \sim \sqrt{2\pi n}\left(\frac{n}{e}\right)^n$ as well as $\varepsilon = \mathrm{poly}(n)^{-1}$, we obtain from (82) that $C(K,\varepsilon) = \Omega(\mathrm{poly}(n)^n)$.

Supplementary References

1. El Gamal, A. & Kim, Y.-H. *Network Information Theory* (Cambridge University Press, Cambridge, 2011).

2. Ahlswede, R. *Multi-way communication channels. Second International Symposium on Information Theory: Tsahkadsor, Armenia, USSR, Sept. 2-8, 1971* (1971), 23–52.
3. Liao, H. *Multiple access channels* PhD thesis (Department of Electrical Engineering, University of Hawaii, 1972).
4. Quek, Y. & Shor, P. W. Quantum and superquantum enhancements to two-sender, two-receiver channels. *Physical Review A* **95,** 052329. arXiv: 1701.03813 [quant-ph] (2017).
5. Winter, A. Personal communication. 2019.
6. Nötzel, J. Entanglement-Enabled Communication. Preprint at https://arxiv.org/abs/1910.03796 (2019).
7. Mermin, N. D. Simple unified form for the major no-hidden-variables theorems. *Physical Review Letters* **65,** 3373–3376 (1990).
8. Peres, A. Incompatible results of quantum measurements. *Physics Letters A* **151,** 107–108 (1990).
9. Aravind, P. K. A simple demonstration of Bell's theorem involving two observers and no probabilities or inequalities. Preprint at https://arxiv.org/abs/quant-ph/0206070 (2002).
10. Brassard, G., Broadbent, A. & Tapp, A. Quantum Pseudo-Telepathy. *Foundations of Physics* **35,** 1877–1907. arXiv: quant-ph/0407221 (2005).
11. Calvo, E., Palomar, D. P., Fonollosa, J. R. & Vidal, J. On the Computation of the Capacity Region of the Discrete MAC. *IEEE Transactions on Communications* **58,** 3512–3525 (2010).
12. Hsieh, M.-H., Devetak, I. & Winter, A. Entanglement-Assisted Capacity of Quantum Multiple-Access Channels. *IEEE Transactions on Information Theory* **54,** 3078–3090. arXiv: quant-ph/0511228 (2008).
13. Winter, A. The capacity of the quantum multiple-access channel. *IEEE Transactions on Information Theory* **47,** 3059–3065. arXiv: quant-ph/9807019 (2001).
14. Yard, J., Hayden, P. & Devetak, I. Capacity theorems for quantum multiple-access channels: classical-quantum and quantum-quantum capacity regions. *IEEE Transactions on Information Theory* **54,** 3091–3113. arXiv: quant-ph/0501045 (2008).
15. Czekaj, L. & Horodecki, P. Purely Quantum Superadditivity of Classical Capacities of Quantum Multiple Access Channels. *Physical Review Letters* **102,** 110505 (2009).
16. Qi, H., Wang, Q. & Wilde, M. M. Applications of position-based coding to classical communication over quantum channels. *Journal of Physics A: Mathematical and Theoretical* **51,** 444002. arXiv: 1704.01361 [quant-ph] (2018).
17. Sen, P. A one-shot quantum joint typicality lemma. Preprint at https://arxiv.org/abs/1806.07278 (2018).
18. Cleve, R. & Mittal, R. *Characterization of binary constraint system games. International Colloquium on Automata, Languages, and Programming* (2014), 320–331. arXiv: 1209.2729 [quant-ph].
19. Slofstra, W. The set of quantum correlations is not closed. *Forum of Mathematics, Pi* **7,** e1. arXiv: 1703.08618 [quant-ph] (2019).
20. Slofstra, W. & Vidick, T. Entanglement in Non-local Games and the Hyperlinear Profile of Groups. *Annales Henri Poincaré* **19,** 2979–3005. arXiv: 1711.10676 [quant-ph] (2018).
21. Zhang, Z. Estimating Mutual Information Via Kolmogorov Distance. *IEEE Transactions on Information Theory* **53,** 3280–3282 (2007).

22. Buhler, J. & Wunder, G. A Note on Capacity Computation for the Discrete Multiple Access Channel. *IEEE Transactions on Information Theory* **57,** 1906–1910 (2011).

23. Arora, S., Lund, C., Motwani, R., Sudan, M. & Mario Szegedy. *Proof verification and hardness of approximation problems. Proceedings of the 33rd Annual Symposium on Foundations of Computer Science* (1992), 14–23.

24. Dinur, I. The PCP Theorem by Gap Amplification. *Journal of the ACM* **54,** 12 (June 2007).

25. Vazirani, V. V. *Approximation Algorithms* (Springer-Verlag, Berlin, Heidelberg, 2001).

26. Arora, S. & Safra, S. *Probabilistic checking of proofs; a new characterization of NP. Proceedings of the 33rd Annual Symposium on Foundations of Computer Science* (1992), 2–13.

27. Feige, U., Goldwasser, S., Lovasz, L., Safra, S. & Szegedy, M. *Approximating clique is almost NP-complete. Proceedings of the 32nd Annual Symposium of Foundations of Computer Science* (1991), 2–12.

28. Håstad, J. Some Optimal Inapproximability Results. *Journal of the ACM* **48,** 798–859 (July 2001).

29. Feige, U. A Threshold of Ln N for Approximating Set Cover. *Journal of the ACM* **45,** 634–652 (1998).

30. Khot, S. & Saket, R. *A 3-query non-adaptive PCP with perfect completeness. Proceedings of the 21st Annual IEEE Conference on Computational Complexity* (2006), 11 pp.–169.

31. Arimoto, S. An algorithm for computing the capacity of arbitrary discrete memoryless channels. *IEEE Transactions on Information Theory* **18,** 14–20 (1972).

32. Blahut, R. Computation of channel capacity and rate-distortion functions. *IEEE Transactions on Information Theory* **18,** 460–473 (1972).

33. Impagliazzo, R. & Paturi, R. *Complexity of k-SAT. Proceedings of the Fourteenth Annual IEEE Conference on Computational Complexity* (1999), 237–240.
